# Investigation of the Prognostic Value of Novel Laboratory Indices in Patients with Sepsis in an Intensive Care Unit: A Retrospective Observational Study

**DOI:** 10.3390/jcm14196765

**Published:** 2025-09-24

**Authors:** Korhan Kollu, Betul Cigdem Yortanli, Ayse Nur Cicek, Emre Susam, Nalan Karakas, Muhammet Cemal Kizilarslanoglu

**Affiliations:** 1 Department of Internal Medicine, Division of Intensive Care Medicine, University of Health Sciences, Konya City Hospital, Akabe, Adana Çevre Yolu Cd. No:135/1, 42020 Karatay, Türkiye; 2 Department of Internal Medicine, University of Health Sciences, Konya City Hospital, 42020 Konya, Türkiye; drbetul85@gmail.com (B.C.Y.); aysenurcamgoz24@gmail.com (A.N.C.); emresusammd@gmail.com (E.S.); aktas.nln.na@gmail.com (N.K.); 3 Department of Internal Medicine, Division of Geriatrics, University of Health Sciences, Konya City Hospital, 42020 Konya, Türkiye; drcemalk@yahoo.com.tr

**Keywords:** albumin, biomarkers, C-reactive protein, prognosis, sepsis

## Abstract

**Background:** This study aimed to evaluate the prognostic value of some novel laboratory indices in intensive care unit (ICU)-hospitalized sepsis patients. **Methods:** This retrospective, observational study included 400 patients with sepsis. The indices studied were the C-reactive protein/albumin ratio (CAR), hemoglobin, albumin lymphocyte, and platelet (HALP) score, lymphocyte/monocyte ratio (LMR), prognostic nutritional index (PNI), systemic immune inflammatory index (SII), vitamin B12xC-reactive protein index (BCI), systemic inflammatory response index (SIRI), and platelet/lymphocyte ratio (PLR). The predicting effects of these indices in ICU mortality, along with other clinical outcomes, were investigated. **Results:** The median age of the study population was 73 (18–95) years and 51.6% were males. The ICU mortality rate was 51.7%. Deceased patients with sepsis had an increased age and high APACHE II and SOFA scores compared to the survivors (*p* < 0.05 for all). In the multivariate logistic regression analysis, age (HR = 1.069, *p* = 0.038 in Model 1 vs. HR = 1.053, *p* = 0.001 in Model 2), SOFA score (HR = 2.145, *p* < 0.001 in Model 1 vs. HR = 1.740, *p* < 0.001 in Model 2), phosphorus levels (in Model 1, HR = 0.608, *p* = 0.037), and CAR (in Model 2, HR = 1.012, *p* = 0.023) were independent associated factors for ICU mortality. According to the ROC analyses, the SOFA (AUC = 0.879, *p* < 0.001) and APACHE II (AUC = 0.769, *p* < 0.001) scores showed high accuracy in predicting ICU mortality, while the PNI (AUC = 0.675, *p* < 0.001), CAR (AUC = 0.609, *p* < 0.001), and the BCI (AUC = 0.648, *p* < 0.001) showed limited accuracy. However, the HALP score did not reach a significant level in predicting ICU mortality (*p* = 0.067). **Conclusions:** Excluding the HALP score, the new laboratory indices mentioned above may be prognostic markers for predicting clinical outcomes in intensive care units for patients with sepsis. However, these indices need to be supported by larger patient populations.

## 1. Introduction

On 26 May 2017, the World Health Organization (WHO) and the World Health Assembly (WHA) declared jointly that sepsis was a global health priority, as it had a dramatically increasing annual rate of 5–13% over the last ten years [1]. Either community or health-care-acquired, sepsis ensues with fatal organ dysfunction or failure because of a dysregulated host response to infection [2]. Its actual burden is estimated as 31 million cases every year, of which 6 million deaths in 2017 [1,3]. Recent data indicate that 20% of all global deaths have been sepsis-related, and global age-standardized mortality in 2017 was 148.1 deaths/100,000 population [4,5].

A wide range of molecules, including acute phase reactants, chemokines, cytokines, damage-associated molecular patterns, metabolites and changes in gene expression, have been investigated as potential biomarkers for early recognition, risk stratification, prognostication and patient management of sepsis and organ dysfunction, either alone or in combined forms as scoring systems [6,7,8,9,10,11,12,13]. Over the last decade, the number of studied diagnostic and prognostic biomarkers, such as C-reactive protein (CRP), the Acute Physiology and Chronic Health Evaluation II (APACHE II), and Sepsis-related Organ Failure Assessment (SOFA) scores, has increased [13]. The APACHE II score, which was developed for several research and clinical audit applications, is frequently utilized for measuring disease severity in critically ill patients. The APACHE II score ranges from 0 to 71, with a higher score indicating a poorer prognosis in this population [14]. After its introduction into use in the early 1990s, the SOFA score has been incorporated into various critical care practices and is currently extensively utilized for the routine assessment of acute morbidity in critical care settings. Although it was initially offered as a population-based understanding tool of acute morbidity among patients hospitalized in intensive care units (ICUs), its usage has significantly expanded recently. With the introduction of updated definitions, it is currently a crucial parameter to diagnose sepsis syndrome at the individual patient level [15,16]. The score from SOFA may range from 0 to 24 points, with higher scores indicating poorer prognosis and higher mortality rates. The CRP/albumin ratio (CAR) is a ratio calculated by dividing CRP by albumin levels [17]. Some clinical studies have shown that its levels on ICU admission may be a new potential independent risk factor for mortality in sepsis patients [18]. However, none alone has the targeted properties for routine use in daily clinical practice [13]. To this end, there is still an unmet need for a viable marker that is reliable, reproducible, cost-effective, and easy to measure, even in health care settings with low income or inadequate laboratory facilities, for screening sepsis patients at a high risk for death [19,20].

The Hemoglobin, Albumin, Lymphocyte, Platelet (HALP) score has recently emerged as a new immune-nutritional biomarker in various cancers [21,22]. Since its first publication in 2015, a low pretreatment HALP score has been determined as a reliable, cost-effective negative prognostic biomarker in patients with solid malignancies [21,22,23]. Accumulated data about the negative prognostic value of pretreatment HALP scores in many oncologic diseases elicited investigation of HALP scores in non-malignant conditions, such as acute/subacute cerebral venous sinus thrombosis, acute ischemic stroke, antineutrophil cytoplasmic antibody-associated vasculitis, sleeve gastrectomy, and several comorbidities [22,24,25,26,27]. The formula “hemoglobin (g/L) × albumin (g/L) levels × lymphocyte count (/L)/platelet count (/L)” is used to calculate the HALP score.

The systemic inflammation response index (SIRI), which is calculated by dividing the neutrophil and monocyte counts by the lymphocyte count, has been found to be closely related to the prognosis of multiple diseases [28,29,30,31]. A study by Zhang et al. found that elevated SIRI was associated with an increased risk of sepsis in stroke patients [28].

The systemic immune-inflammation index (SII), which is based on platelet, lymphocyte and neutrophil counts, was introduced [32]. Given the robust relationship between thrombosis and inflammation, incorporating three independent blood-tested biomarkers into a single index has led to the SII index being described as a predictor of adverse outcomes in various conditions, including oncology, cardiovascular diseases, and intracerebral hemorrhage [33,34,35].

The Prognostic Nutritional Index (PNI), calculated from albumin and lymphocyte levels, has shown potential for predicting mortality in various conditions, cardiovascular diseases, liver diseases, and chronic kidney diseases [36,37].

However, there are few studies on the prognostic value of PNI in patients with sepsis [38].

In patients with sepsis, thrombocytopenia and lymphopenia may develop, albumin levels may decrease as a negative acute phase reactant, and CRP may increase. For these reasons, indices such as the HALP score, BCI, PNI, and CAR may show variability. Numerous laboratory indices, such as the HALP score, CAR, vitamin B12 and CRP index (BCI), SIRI, SII, PNI also show that they can be used in some clinical situations for predicting prognosis. Their predicting power in sepsis patients, to our knowledge, is limited and still waiting to be explored in clinical studies. Thus, the current study aimed to evaluate the prognostic value of some novel laboratory indices in patients with sepsis hospitalized in ICUs.

## 2. Materials and Methods

In this observational, retrospective study, 1900 patients (>18 years of age) admitted to the ICU of the Internal Medicine Department of Konya City Hospital between 1 April 2021 and 1 April 2023 were screened and the medical records of patients diagnosed with sepsis were reviewed. As stated in the sepsis survival guidelines, sepsis is defined as life-threatening acute organ failure resulting from the host’s abnormal response to infection. The SOFA score is used to assess organ failure [2]. Sepsis was defined as an increase of 2 or more points in the SOFA score in the presence of infection [16]. Patients with solid organ tumors, hematologic malignancies, or who were under chemotherapy were excluded. Ethical approval of this study was granted by KTO Karatay University Faculty of Medicine Ethics Committee with the date and number 27.04.2023-2023/017. Due to its retrospective design, informed consent from the participants was not needed for this study. The medical records of 400 patients were reviewed for demographic characteristics, co-morbidities, underlying pathologies, and reasons for ICU admission, and data about the APACHE II and SOFA scores, hemogram, biochemical and serologic parameters, CRP, procalcitonin, and indices were collected. The laboratory parameters were collected from the blood samples taken on the first day of ICU admission. The hospital and ICU length of stay (LOS) was calculated according to admission and discharge dates. ICU and hospital clinical outcomes were recorded from the patient’s files in the hospital computer system (dead or alive).

The indices investigated for the predictive prognostic value were the platelet/lymphocyte ratio (PLR), CAR, HALP score, BCI, lymphocyte/monocyte ratio (LMR), systemic immune-inflammation index (SII), systemic inflammatory response index (SIRI), and prognostic nutritional index (PNI).

The BCI, LMR, PLR, SII, PNI, and SIRI are calculated according to the complete blood count (CBC) results obtained from the peripheral blood samples by the given formulas [8,10,32,39,40,41]:

BCI = Vitamin B12 (pmol/L) × CRP (mg/L).

LMR = Lymphocyte count (/mm^3^)/monocyte count (/mm^3^).

PLR = Platelet count (/mm^3^)/Lymphocyte count (/mm^3^).

SII = Platelet count (/mm^3^) × neutrophil count (/mm^3^)/lymphocyte count (/mm^3^).

SIRI = [neutrophil count (/mm^3^) × monocyte count (/mm^3^)]/lymphocyte count (/mm^3^)]).

PNI = 10 × albumin (g/dL) + 0.005 × total lymphocyte count (/mm^3^).

### Statistical Analysis

Each patient’s LOS in the hospital and ICU was collected. Patient data were grouped into two groups according to the outcomes: survivors of sepsis and deceased patients. Age, APACHE II, SOFA, blood results, and indices of the groups were compared. A correlation analysis was performed between each index and clinical/laboratory data. In the correlation analysis, the strength of the relationship was interpreted according to the following criteria: weak (0–0.3 or 0 to −0.3), moderate (0.3–0.5 or −0.3 to −0.5), strong (0.5–0.7 or −0.5 to −0.7), and very strong (>0.7 or <−0.7). A correlation coefficient closer to ±1 indicates a stronger linear relationship, whereas values closer to 0 indicate a weaker relationship. The specificity and sensitivity of the CAR, HALP, APACHE II, SOFA scores, BCI, and PNI in predicting ICU mortality was determined using the Receiver Operating Characteristic (ROC) curve analysis. Mortality-related parameters were investigated using binary logistic regression analysis. In Model 1, mortality-related parameters, which were determined in the univariate analyses (acute pancreatitis, age, SOFA, ferritin, international normalized ratio (INR), CAR, BCI, PNI, calcium, phosphorus, magnesium, T3, T4, HbA1c), were included in multivariate binary logistic analysis. In Model 2, multivariate binary logistic analysis included mortality-related parameters determined in univariate analyses (acute pancreatitis, age, SOFA, ferritin, INR, CAR, BCI, PNI). For both models, the backward regression method was used.

The normality of data was tested using the Kolmogorov–Smirnov test, histogram, and coefficient of variation. Numerical variables which were distributed normally were expressed as mean with standard deviation, whereas those which were not distributed normally were expressed as median and minimum and maximum. Categorical variables were expressed as number (percentage). The independent groups were compared using a student’s T-test or Mann–Whitney U test, where appropriate. Categorical data between independent groups were compared using the chi-squared or Fisher’s exact tests. The level of significance was set at a *p* value of <0.05. Data analyses were performed using the IBM SPSS Statistics for Windows (version 21.0; IBM Corp., Armonk, NY, USA).

## 3. Results

The age of the cohort had a median of 73 years (range, 18–95 years). Of the cohort, 51.6% (*n* = 206) were males, and 48.3% (*n* = 197) were discharged from the hospital. The APACHE II and SOFA scores had a median of 26 (range, 5–55) and 7 (range, 2–19), respectively. The participants had more than one comorbidity, and hypertension (48.0%, *n* = 192), diabetes mellitus (39%, *n* = 152), and acute renal failure (39%, *n* = 156) were the leading ones (Table 1).

**Figure 1:**
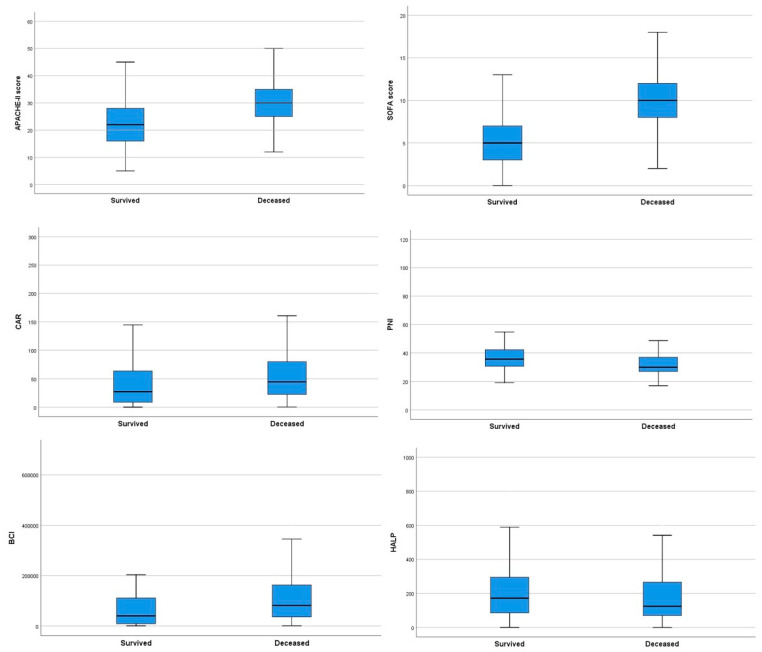
Comparison of prognostic scores and biomarkers between surviving and deceased patients using box plots.

Deceased patients with sepsis had increased age, and high APACHE II and SOFA scores when compared to survivors (*p* < 0.05, Table 1). In the CBC, the deceased patients had significantly lower platelet (*p* = 0.006) and lymphocyte (*p* = 0.007) counts. The aspartate aminotransferase, total bilirubin, direct bilirubin, procalcitonin, ferritin, vitamin B12, INR, and D-dimer levels of deceased patients were significantly higher than those of discharged patients (*p* < 0.001). The groups significantly differed in blood calcium, phosphorus, and magnesium levels. CRP levels were higher (*p* = 0.004), whereas fibrinogen and albumin levels were significantly lower in the deceased patients (*p* < 0.05, Table 1). When the two groups were compared, the surviving patients had lower CAR and BCI, shorter stays at the ICU, and higher prognostic nutritional index values (*p* < 0.001 for all). The comparisons between two patient groups are presented in Table 1. The comparison of APAHE-II scores, SOFA scores, HALP scores, BCI, PNI, and CAR indices between the groups of surviving and deceased patients is shown in Figure 1.

According to the correlation analysis performed between the indices and clinical/laboratory parameters (Table S1), besides albumin and CRP levels, CAR was positively correlated with procalcitonin (strong correlation) and fibrinogen (moderate correlation), yet it was negatively associated with blood lipids (moderate correlation with LDL-C and HDL-C; weak correlation with triglycerides) (*p* < 0.01 for all). The HALP score was positively correlated with hemoglobin (weak correlation), mean platelet volume (weak correlation), lymphocyte count (moderate correlation), monocyte count (weak correlation), total bilirubin (weak correlation), albumin (weak correlation) (*p* < 0.01 for all), direct bilirubin (weak correlation), and sodium (weak correlation) (*p* < 0.05 for all). High HALP scores were associated with low CRP (weak correlation), short hospital (weak correlation) and ICU stays (weak correlation) (*p* < 0.01 for all), and low fibrinogen levels (weak correlation) (*p* < 0.05).

Among the indices, the BCI was the only one correlated with age (weak correlation) (rho = −0.177; *p* < 0.01). A high BCI was moderately to weakly correlated with high ferritin (moderate correlation), INR (weak correlation), and D-dimer levels (weak correlation), whereas low albumin levels demonstrated a strong correlation (*p* < 0.01). LMR negatively correlated with white blood cell (weak correlation) and neutrophil counts (weak correlation) (*p* < 0.01 for all). The inflammatory markers SII and SIRI positively correlated with white blood cell (strong correlation), neutrophil (strong correlation), and monocyte (weak correlation with SII and strong correlation with SIRI) counts (*p* < 0.01 for all). The PNI showed positive correlations with hemoglobin (moderate correlation), calcium (moderate correlation), and blood lipids (weak correlation with triglyceride, moderate correlations with HDL-C and LDL-C); however, there were negative correlations with CRP (moderate correlation) and procalcitonin (moderate correlation) (*p* < 0.01 for all). The results of correlation analyses are presented in Table S1.

**Figure 2:**
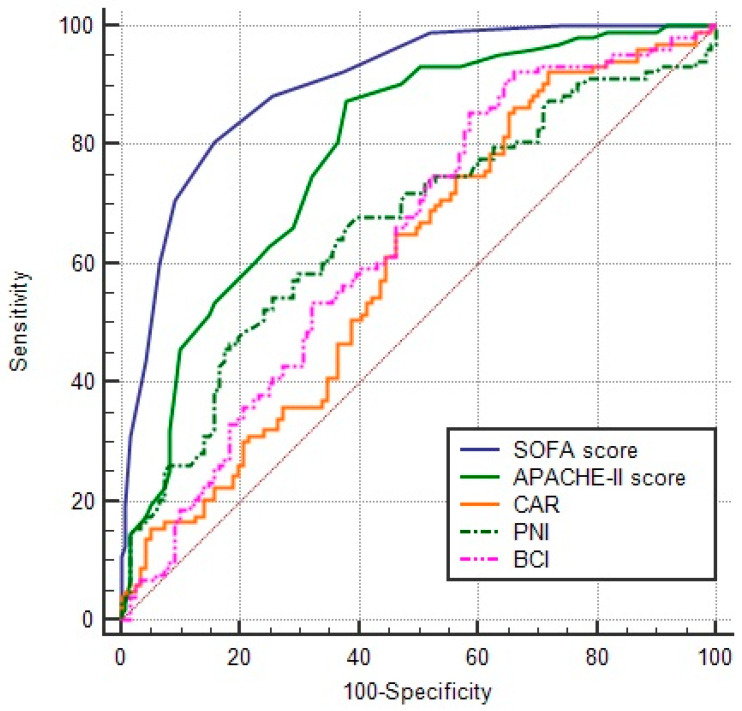
This figure shows the comparisons of the ROC curves between the prognostic indices for mortality (SOFA vs. APACHE-II *p* < 0.001, SOFA vs. CAR *p* < 0.001, SOFA vs. PNI *p* < 0.001, SOFA vs. BCI *p* < 0.001, APACHE-II vs. CAR *p* < 0.001, APACHE-II vs. PNI *p* = 0.012, APACHE-II vs. BCI *p* = 0.001, CAR vs. PNI *p* = 0.134, CAR vs. BCI *p* = 0.176, PNI vs. BCI *p* = 0.578).

According to the ROC analysis, the SOFA and APACHE II scores had higher diagnostic power than the PNI, CAR, HALP, and BCI (Table 2, Figure 2). However, the HALP score did not reach the significance level of the predictive marker in ROC analysis (*p* = 0.067). Among the six indices, the BCI had the highest sensitivity with 87.2% but the lowest specificity. While the SOFA and APACHE II scores were comparable in sensitivity, the SOFA scores had higher specificity than APACHE II (Table 2). The multivariate logistic regression analysis indicated that age, SOFA score, and phosphorus levels effectively affected mortality (Model 1). In Model 2, age, SOFA score, and CAR were determined to affect mortality (Table 3).

## 4. Discussion

The actual burden of sepsis is still unknown. Yet, the most recent rates are estimations relying on a systematic review extrapolating from published national or local population estimates to the global population [3]. However, the global estimates partially reflect actual data about sepsis burden in low- and middle-income countries. In line with the sepsis resolution adopted by the WHA in 2017 [1], we investigated whether the APACHE-II, SOFA score, HALP score, SIRI, SII, PNI, and BCI might have prognostic biomarkers in sepsis patients. Conversely, together with older age, higher levels of APACHE II, SOFA, CAR, BCI, and PNI were more pronounced in deceased patients. We determined that age, SOFA score, CAR, and phosphorus levels were mortality-related parameters; however, the specificity and sensitivity levels of CAR, PNI, and BCI alone were not satisfactory for their biomarker designation for sepsis.

Due to ongoing intense interest in defining biomarkers for sepsis and septic shock, more than 250 biomarkers are currently used for prognostic, predictive, and diagnostic purposes [13,42]. Of them, 100 (39%) were assessed for prognostic, and 89 (34%) were evaluated for both diagnostic and prognostic purposes [13]. However, defining a biomarker with an accurate prognostic value is still an unmet need due to poor sensitivity, poor predictive value, and a wide variety of sepsis-related pathways [2,13,43]. In the 2021 Surviving Sepsis Campaign (SSC) guidelines, it is strongly recommended that the quick SOFA (qSOFA), commonly used in clinical evaluation for sepsis, should not be used as a single screening tool due to its poor sensitivity [2]. Moreover, a single measurement of lactate levels, another proposed screening tool for sepsis, had poor predictive value, and lactate might be elevated in various conditions [2]. Hence, it is proposed to measure the combination of biomarkers to overcome the shortcomings of a single biomarker so that it may involve different sepsis-related pathways [13].

Clinicians faced abnormal responses of the host immune system leading to multiple organ failure, pneumonia, septic shock and death during the COVID-19 pandemic [17,44,45]. Suggested biomarkers for poor prognosis included procalcitonin, CRP, the neutrophil-to-lymphocyte ratio, albumin, apolipoproteins, D-dimer, and ferritin [45]. CAR was reported as a novel biomarker in laboratory-confirmed cases (diagnostic accuracy, 89.3%; optimal cut-off, 0.475) [44]. A systematic review and meta-analysis on 22 studies carried out on coronavirus disease 2019 (COVID-19) indicated that high CAR correlated with severe disease or mortality [45]. The predictive performance of CAR was first studied on 6414 sepsis patients among 30 potential predictors, including age, high-density lipoprotein, ferritin, SOFA score, lymphocyte, and neutrophil counts [17]. The subgroup analysis between CAR (mean 17.5 for both groups) and overall survival (OS) revealed that higher CAR levels were significantly associated with lower OS in each subgroup. CAR was an independent prognostic marker for in-hospital mortality in sepsis patients. Moreover, it is reported that the CAR-based risk-prediction nanogram had high clinical application value in the stratification of sepsis patients and in initiating goal-directed treatment [17]. In the current literature, data on the prognostic value of CAR in patients with sepsis is increasing [18,46].

In the present study, higher CAR was found in deceased patients with a higher median than the cut-off value (Table 1 and Table 2). Although CAR was not determined to stand alone as a prognostic biomarker, a combination of patient age, CAR level, SOFA score, APACHE-II score, and clinical state might be considered a reliable guide for clinicians as the first three were determined to be significantly associated with mortality (Table 2). As the SSC Guidelines 2021 recommended, a relevant combination of prognostic biomarkers may have a higher predictive value for sepsis outcomes.

The SOFA score has significant potential for early diagnosis of sepsis and dynamic monitoring of patient condition. Previous studies have shown that clinicians can use the SOFA score to evaluate organ function in patients with sepsis [47]. The score evaluates the respiratory, hematological, hepatic, circulatory, neurological and renal functions of subjects and facilitates the assessment of individual organ function through the scale. A controlled study involving 1782 intensive care patients with sepsis found that the SOFA score had an area under the curve (AUC) value of 0.879 for predicting mortality in septic intensive care patients, and that SOFA scores were significantly higher in deceased patients than in survivors (*p* < 0.05) [48]. These findings are consistent with those of our study.

The APACHE II scoring system requires an assessment to be carried out within 24 h of a patient being admitted to an intensive care unit, and it is widely used in clinical practice. The score obtained from this scale provides a quantitative assessment of the patient’s condition and can be used to predict mortality. Studies conducted on sepsis patients show that the higher the APACHE II score, the higher the mortality rate among treated patients [49]. This indicates that the APACHE II score is an effective predictor of the prognosis of septic patients. Li et al. demonstrated that using the APACHE II scale yielded an area under the curve (AUC) of 0.806 in assessing poor prognosis in septic patients [50]. This is consistent with the findings of other researchers and our own study. However, the APACHE II score has several drawbacks, including a large number of assessment indicators and a lengthy assessment process, which may render it unsuitable for critically ill patients.

In our cohort of septic patients, the differential prognostic relevance of these indices deserves careful interpretation. The CAR, by integrating an acute-phase reactant (CRP) with a marker of nutritional and inflammatory reserve (albumin), has strong theoretical plausibility and pathophysiological rationale in the context of sepsis, where dysregulated host response and catabolic stress coexist. In contrast, indices such as HALP or BCI are derived from broader oncologic or surgical populations, and their translational grounds in sepsis remain relatively weak. While they may capture aspects of baseline nutritional or comorbidity burden, their predictive value is less consistent when applied to critically ill, infection-driven settings. Importantly, the clinical utility of any prognostic marker in sepsis is limited unless it can inform therapeutic decision-making or patient stratification. Future studies should therefore aim to determine not only the prognostic accuracy of these indices but also whether they can guide individualized interventions, such as targeted nutritional support or immunomodulatory strategies.

In our study, low serum phosphorus levels were independently associated with increased mortality. Hypophosphatemia may worsen outcomes by impairing cellular energy metabolism, reducing diaphragmatic contractility, and contributing to cardiovascular instability. It may also reflect malnutrition, sepsis-related redistribution, or refeeding syndrome, all of which are linked to poor prognosis [51].

The present study has a number of limitations. Firstly, the retrospective nature of the study design means that bias is an inherent problem. Secondly, some data about covariates associated with sepsis were missing in patient files, which limited the study database. Thirdly, a single measurement of laboratory tests was performed on samples obtained at different time frames, which might affect the laboratory results. Fourthly, we did not stratify patients according to the cause of their sepsis (e.g., bacterial, viral or parasitic), which may limit how widely our findings can be applied. Fifth, proinflammatory cytokine and interleukin levels (TNF-α, IL-6, etc.) were not routinely measured and therefore were not used in our study. Lastly, patient files were reviewed at (2–3) different time frames, which might cause selection bias. Therefore, the findings of the current study should be interpreted with caution and confirmed by conducting a large-scale prospective study. Furthermore, the results are primarily applicable to populations with similar demographic and clinical characteristics.

## 5. Conclusions

In septic patients, CAR, age, and the SOFA score emerged as key prognostic factors, whereas indices such as HALP and the BCI showed limited applicability. Established severity scores, particularly SOFA and APACHE II, consistently outperformed newer indices. Among the biomarkers evaluated, CAR was the only one with consistent biological plausibility in sepsis, while others remain in need of exploration and require further validation.

## Figures and Tables

**Table 1:** Comparisons of general characteristics, clinical and laboratory parameters between deceased patients and survivors.

Parameters	All *n* = 400 (%)	Deceased Patients *n* = 207	Survivors *n* = 193	*p*-Value
		*n* (%)	*n* (%)	
Sex				
Male	206 (51.5)	109 (52.7)	97 (50.3)	0.632
Female	194 (48.5)	98 (47.3)	96 (49.7)	0.632
Comorbidities				
Diabetes mellitus	156 (39.0)	74 (35.7)	82 (42.5)	0.167
Hypertension	192 (48.0)	94 (45.4)	98 (50.8)	0.283
Dementia	53 (13.3)	30 (14.5)	23 (11.9)	0.448
Chronic renal failure	69 (17.3)	36 (17.4)	33 (17.1)	0.938
Acute renal failure	156 (39.0)	87 (42.0)	69 (35.8)	0.198
Gastrointestinal bleeding	12 (3.0)	9 (4.3)	3 (1.6)	0.102
Acute pancreatitis	11 (2.8)	2 (1.0)	9 (4.7)	0.023
COPD	62 (15.5)	36 (17.4)	26 (13.5)	0.279
Asthma	34 (8.5)	17 (8.2)	17 (8.8)	0.831
CAD	119 (29.8)	61 (29.5)	58 (30.1)	0.899
CHF	81 (20.3)	37 (17.9)	44 (22.8)	0.221
	Median (Min–Max)	Median (Min–Max)	Median (Min–Max)	*p*-value
Age, years	73.0 (18.0–95.0)	75.0 (21.0–95.0)	69.0(18.0–94.0)	<0.001
APACHE II	26.0 (5.0–55.0)	30.0 (12.0–55.0)	22.0 (5.0–53.0)	<0.001
SOFA score	7.0(0.0–19.0)	10.0 (2.0–19.0)	5.0 (0.0–14.0)	<0.001
Complete blood count				
WBC, 10^3^/µL	11,520.0 (50.0–172,240.0)	12,020.0 (50.0–172,240.0)	11,050.0 (110.0–90,460.0)	0.289
Hemoglobin, g/dL	10.5 (1.7–20.9)	10.3 (5.6–2.9)	10.8 (1.7–18.2)	0.078
MCV, fL	89.2 (63.2–140.8)	89.3 (70.0–129.1)	89.1 (63.2–140.8)	0.330
Platelets, 10^3^/µL	191.0 (3.0–714.0)	164.0 (3.0–1714.0)	209.0 (6.0–613.0)	0.006
MPV, fL	10.8 (0.0–14.8)	11.0 (0.0–14.8)	17.0 (0.0–14.4)	0.133
Neutrophil, 10^3^/µL	9300 (0.0–73,200.0)	9890.0 (0.0–44,110.0)	8910.0 (1.8–73,200.0)	0.316
Lymphocyte, 10^3^/µL	880.0 (0.0–15,090.0)	810.0 (0.0–15,090.0)	990.0 (2.4–4990.0)	0.007
Monocyte, 10^3^/µL	600.0 (0.0–123,810.0)	570.0 (0.0–123,810.0)	650.0 (0.6–3190.0)	0.172
Blood chemistry and serology				
Plasma glucose, mg/dL	141.5(36.0–996.0)	138.5 (36.0–755.0)	143.0 (49.0–996.0)	0.186
Creatinine, mg/dL	1.4 (0.20–8.05)	1.5 (0.2–8.1)	1.3 (0.2–7.4)	0.118
Uric acid, mg/dL	6.7 (1.20–18.1)	6.6 (1.2–17.8)	6.7 (1.6–18.1)	0.752
AST, U/L	32.0 (5.0–13936.0)	38.0 (6.0–13936.0)	26.0 (5.0–4788.0)	<0.001
ALT, U/L	20.5 (5.0–5787.0)	22.0 (5.0–5787.0)	18.0 (5.0–2166.0)	0.027
LDH, U/L	338.0 (33.5–13,393.0)	410.0 (33.5–13,393.0)	312.0 (113.0–5599.0)	<0.001
Total bilirubin, mg/dL	0.8 (0.1–29.8)	1.0 (0.2–29.8)	0.7 (0.1–20.7)	<0.001
Direct bilirubin, mg/dL	0.3 (0.1–21.3)	0.4 (0.1–21.3)	0.3 (0.1–16.6)	<0.001
LDL-cholesterol, mg/dL	58.0 (3.9–218.0)	49.0 (3.9–167.0)	68.5 (3.9–218.0)	<0.001
HDL-cholesterol, mg/dL	28.0 (3.0–86.0)	25.0 (3.0–86.0)	29.5 (7.0–81.0)	0.002
Triglycerides, mg/dL	139.0 (34.0–722.0)	140.0 (46.0–718.0)	135.0 (34.0–722.0)	0.296
Albumin, g/dL	2.8 (1.4–5.2)	2.6 (1.4–4.2)	3.1 (1.4–5.2)	<0.001
Calcium, mg/dL	8.2 (4.7–13.9)	8.0 (4.7–13.6)	8.2 (5.2–13.9)	<0.001
Phosphorus, mg/dL	4.0 (1.0–15.6)	4.2 (1.0–15.6)	3.6 (1.0–8.8)	<0.001
Magnesium, mg/dL	2.0 (1.0–5.6)	2.1 (1.0–3.8)	1.9 (1.1–5.6)	0.006
Sodium, mmol/L	137.0 (110.0–185.0)	138.0 (119.0–164.0)	136.0 (110.0–185.0)	0.118
Potassium, mmol/L	4.4 (1.9–8.6)	4.4 (2.2–8.6)	4.3 (1.9–6.4)	0.073
C-reactive protein, mg/L	104.8 (0.6–458.0)	114.4 (2.1–427.4)	83.9 (0.6–458.0)	0.004
PRC, µg/L	1.0 (0.0–100.0)	1.6 (0.0–100.0)	0.4 (0.0–100.0)	<0.001
TSH, mU/L	1.2 (0.0–57.0)	1.2 (0.0–21.6)	1.3 (0.0–57.0)	0.855
T3, ng/L	1.4 (0.4–10.3)	1.3 (0.4–10.3)	1.5 (0.4–6.4)	0.007
T4, ng/L	11.3 (0.6–22.8)	11.1 (2.6–22.8)	11.4 (0.6–22.6)	0.202
Ferritin, µg/L	582.0 (3.9–31,069.0)	839.0 (35.0–31,069.0)	430.0 (3.9–8601.0)	<0.001
Folate, µg/L	6.0 (1.2–20.0)	6.6 (1.2–20.0)	5.7 (1.3–20.0)	0.707
Vitamin B12, pmol/L	610.0 (100.0–4000.0)	744.0 (100.0–4000.0)	490.0 (100.0–2000.0)	<0.001
HbA1c, %	6.4 (4.4–18.8)	6.0 (4.4–11.1)	6.5 (4.7–18.8)	0.035
Vitamin D, ng/L	6.1 (3.0–47.3)	7.1 (3.0–34.4)	5.6 (3.0–47.3)	0.731
INR	1.4 (0.9–20.0)	1.5 (1.0–20.0)	1.3 (1.0–7.2)	<0.001
Fibrinogen, g/L	3.9 (0.3–13.1)	3.6 (0.3–11.6)	4.0 (1.1–13.1)	0.011
D-dimer, mg/L	4.8 (0.2–74.8)	5.9 (0.2–74.8)	3.9 (0.4–35.2)	<0.001
Indices				
CAR	38.1 (0.2–267.2)	44.7 (0.5–267.2)	27.4 (0.2–158.3)	<0.001
HALP score	146.3 (0.2–10353.4)	125.4 (0.8–10,353.4)	171.8 (0.2–2161.4)	0.067
BCI	55,745.6 (746.0–669,385.2)	81,283.2 (866.8–624,041.8)	40,089.0 (746.0–669,385.2)	<0.001
LMR	1.6 (0.0–1674.4)	1.6 (0.0–57.9)	1.7 (1.1–1674.4)	0.330
PLR	0.2 (0.0–176.7)	0.2 (0.0–73.4)	0.2 (0.0–176.2)	0.904
SII	1839.1 (0.1–2,971,761.6)	1828.0 (0.1–1,443,410.1)	1868.2 (0.1–2,971,761.6)	0.920
SIRI	5729.5 (0.8–16,839,784.2)	5949.9 (0.7–16,839,784.2)	5594.8 (0.5–90,973.6)	0.256
PNI	33.2 (17.1–113.5)	30.1 (17.1–113.5)	35.6 (19.1–63.0)	<0.001
LOS, days				
Hospital LOS	15.0 (0.0–745.0)	17.0 (0.0–745.0)	13.0 (0.0–670.0)	0.493
ICU LOS	6.0 (0.0–126.0)	8.0 (0.0–126.0)	4.0 (0.0–108.0)	<0.001

COPD, chronic obstructive pulmonary disease; CAD, coronary artery disease; CHF, congestive heart failure; APACHE II, Acute Physiology and Chronic Health Evaluation II; SOFA score, sequential organ failure assessment score; WBC, white blood cell; MCV, mean corpuscular volume; MPV, mean platelet volume; AST, aspartate aminotransferase; ALT, alanine aminotransferase; LDH, lactate dehydrogenase; LDL, low molecular weight lipoprotein; HDL, high molecular weight lipoprotein; PRC, procalcitonin; TSH, thyroid stimulating hormone; INR, international normalized ratio; CAR, C-reactive protein/albumin ratio; HALP, hemoglobin, albumin, lymphocytes, platelets; BCI, vitamin B12/C-reactive protein index; LMR, lymphocyte-to-monocyte ratio; PLR, platelet-to-lymphocyte ratio; SII, systemic immune-inflammation index; SIRI, systemic inflammatory response index; PNI, prognostic nutritional index; LOS, length of stay; ICU, intensive care unit.

**Table 2:** The Receiver Operating Curve (ROC) analysis results for the indices predicting intensive care unit mortality.

Parameters	*p*-Value	Cut-Off Value	AUC	Sensitivity	Specificity	PPV	NPV
PNI	<0.001	≤29.7	0.675	49.3	82.3	74.6	60.5
CAR	<0.001	>27.9	0.609	68.5	51.8	60.2	60.7
HALP score	0.067	≤133.2	0.553	54.0	61.5	59.6	55.9
BCI	<0.001	>23,282.4	0.648	87.2	40.0	56.7	77.6
APACHE II score	<0.001	>24	0.769	80.2	65.4	70.4	76.3
SOFA score	<0.001	>7	0.879	79.2	81.9	82.4	78.6

AUC, area under the curve; PPV, positive predictive value; NPV, negative predictive value; PNI, Prognostic nutritional index; CAR, C-reactive protein/albumin ratio; HALP, hemoglobin-albumin-lymphocytes-platelets; BCI, vitamin B12/C-reactive protein index; APACHE II, Acute Physiology and Chronic Health Evaluation II; SOFA score, sequential organ failure assessment score.

**Table 3:** Multivariate (binary logistic) regression analysis of mortality-related parameters.

Mortality-Related Parameters for Models	HR	95% CI		*p*-Value
		Lower Bound	Upper Bound	
Model 1 †				
Age, years	1.069	1.004	1.139	0.038
SOFA score	2.145	1.568	2.935	<0.001
Phosphorus	0.608	0.381	0.969	0.037
Model 2 ‡				
Age, years	1.053	1.021	1.085	0.001
SOFA score	1.740	1.505	2.011	<0.001
CAR	1.012	1.002	1.022	0.023

HR, hazard ratio; CI, confidence interval; SOFA score, sequential organ failure assessment score; CAR, C-reactive protein/albumin ratio. † For Model 1, parameters that were found to be associated with death in univariate analyses (acute pancreatitis, age, SOFA, ferritin, INR, CAR, BCI, PNI, Ca, phosphorus, Mg, T3, T4, and HbA1C) were included in the multivariate binary logistic regression analysis. Backward regression method was used. The last step (step 8) is presented in the table. For this step, Omnibus test *p* < 0.001, Hosmer–Lemeshow *p* = 0.683, and Nagelkerke R square = 0.719. Because there was a high correlation between CAR and the BCI, CAR was not included in the multivariate analysis. ‡ For Model 2, the multivariate binary logistic regression analysis included parameters associated with death in univariate analyses (acute pancreatitis, age, SOFA, ferritin, INR, CAR, BCI, and PNI). The backward regression method was used. The last step (step 4) is presented in this table. For this step, Omnibus test *p* < 0.001, Hosmer–Lemeshow *p* = 0.895, and Nagelkerke R square = 0.613. Because there was a high correlation between CAR and the BCI, the BCI was not included in the multivariate analysis.

## Data Availability

The raw data supporting the conclusions of this article will be made available by the authors on request.

## Supplementary Materials

The following supporting information can be downloaded at: https://www.mdpi.com/article/10.3390/jcm14196765/s1, Table S1: Correlation analysis between clinical/laboratory parameters and indices.
