# Supplementary material for: Investigation of the Prognostic Value of Novel Laboratory Indices in Patients with Sepsis in an Intensive Care Unit: A Retrospective Observational Study
Source: J Clin Med. 2025 Sep 24;14(19):6765. doi: 10.3390/jcm14196765 (PMC12525439; doi:10.3390/jcm14196765)
Supplement: Supplementary file 1 [file jcm-14-06765-s001.zip › jcm-3826734-supplementary.pdf]

Table S1. Correlation analysis between clinical/laboratory parameters and indices.

Parameters	CAR	HALP	BCI	LMR	PLR	SII	SIRI	PNI
Age, years	-0.017	-0.055	-0.177**	-0.033	0.015	0.022	0.019	-0.084
APACHE II	0.128*	-0.008	0.158*	-0.009	-0.060	-0.034	0.010	-0.136**
SOFA	0.197**	-0.026	0.271**	-0.057	-0.088	-0.051	0.078	-0.274**
Complete blood count								
WBC, $10^3/\mu L$	0.123*	0.021	0.207**	-0.320**	0.007	0.554**	0.708**	0.115*
Hemoglobin, g/dL	-0.167**	0.260**	-0.138*	-0.041	0.069	0.156**	0.125*	0.315**
MCV, fL	-0.049	0.096	-0.117	0.086	-0.100*	-0.104*	-0.057	-0.051
Platelet, $10^3/\mu L$	-0.045	-0.465**	-0.069	-0.098	0.560**	0.610**	0.205**	0.209**
MPV, fL	0.068	0.156**	0.067	0.071	-0.187**	-0.152**	-0.042	-0.109*
Neutrophil, $10^3/\mu L$	0.137**	-.0028	0.177**	-0.333**	0.061	0.641**	0.752**	0.051
Lymphocyte, $10^3/\mu L$	-0.117*	0.570**	-0.085	0.263**	-0.580**	-0.242**	-0.039	0.473**
Monocyte, $10^3/\mu L$	-0.096	0.160**	0.030	-0.637**	-0.120*	0.229**	0.685**	0.289**
Blood chemistry and serology								
Plasma glucose, mg/dL	-0.030	0.013	0.005	-0.047	0.070	0.147**	0.098	0.154**
Creatinine, mg/dL	0.112*	0.045	0.131*	-0.124*	-0.048	0.021	0.145**	0.042
Uric acid, mg/dL	0.021	0.095	0.083	-0.096	-0.081	0.031	0.130*	0.136**
AST, U/L	0.104*	0.058	0.245**	-0.047	-0.090	-0.011	0.096	-0.059
ALT, U/L	-0.004	0.070	0.197**	-0.033	-0.056	-0.016	0.059	0.026
LDH, U/L	0.059	0.078	0.175**	-0.020	-0.100*	-0.035	0.077	-0.026
Total bilirubin, mg/dL	0.069	0.198**	0.145*	-0.052	-0.221**	-0.190**	0.040	-0.110*
Direct bilirubin, mg/dL	0.168**	0.125*	0.227**	-0.064	-0.189**	-0.152**	0.052	-0.198**
LDL-C, mg/dL	-0.376**	0.078	-0.244**	-0.014	0.106	0.054	-0.046	0.382**
HDL-C, mg/dL	-0.418**	0.015	-0.352**	0.002	0.172**	0.096	-0.063	0.364**
Triglycerides, mg/dL	0.225**	0.018	0.219**	0.088	-0.051	-0.099	-0.085	-0.086
Albumin, g/dL	-0.516**	0.209**	-0.362**	-0.081	0.100*	0.038	0.024	0.859**
Calcium, mg/dL	-0.250**	0.076	-0.121	-0.053	0.109*	0.099*	0.033	0.491**

Phosphorus, mg/dL	0.050	-0.032	0.119	-0.051	-0.008	0.074	0.117*	-0.039
Magnesium, mg/dL	-0.066	-0.020	-0.027	-0.157**	0.041	0.142**	0.192**	-0.011
Sodium, mmol/L	-0.026	0.107*	-0.137*	0.155**	-0.101*	-0.070	-0.114*	0.013
Potassium, mmol/L	-0.047	-0.063	0.043	-0.176**	0.097	0.137**	0.166**	0.102*
C-reactive protein, mg/L	0.974**	-0.165**	0.756**	-0.008	0.069	0.086	0.087	-0.327**
PRC, μg/L	0.594**	-0.066	0.523**	-0.052	-0.022	0.019	0.088	-0.352**
TSH, mU/L	0.005	-0.011	0.078	0.096	-0.037	0.001	-0.051	0.013
T3, ng/L	-0.171**	0.037	-0.161*	0.012	0.049	-0.016	-0.059	0.303**
T4, ng/L	-0.029	0.020	-0.041	-0.045	0.049	-0.010	-0.015	0.114
Ferritin, μg/L	0.311**	-0.038	0.315**	-0.020	-.0066	-0.073	0.027	-0.251**
Folate, μg/L	-0.122	-0.031	0.105	-0.061	0.085	0.042	0.034	0.218**
Vitamin B12, pmol/L	0.086	-0.054	0.660**	-0.095	-0.009	0.055	0.121	-0.136*
HbA1c, %	-0.043	0.097	-0.064	-0.101	0.011	0.043	0.089	0.142
Vitamin D, ng/L	-0.090	0.018	0.079	0.093	0.025	-0.034	-0.088	0.125
INR	0.282**	-0.030	0.269**	0.000	-0.118*	-0.087	0.038	-0.305**
Fibrinogen, g/L	0.340**	-0.142*	0.151*	0.014	0.185**	0.150*	-0.016	0.028
D-dimer, mg/L	0.292**	0.014	0.236**	-0.090	-0.107	-0.066	0.095	-0.178**
**LOS, days**								
Hospital-LOS	-0.055	-0.172**	0.008	-0.033	0.124*	0.074	-0.033	-0.018
ICU-LOS	0.024	-0.245**	0.025	-0.009	0.181**	0.164**	0.023	-0.164**

CAR, C-reactive protein/albumin ratio; HALP, hemoglobin, albumin, lymphocytes, platelets; BCI, vitamin B12/C-reactive protein index; LMR, lymphocyte-to-monocyte ratio; PLR, platelet-to-lymphocyte ratio; SII, Systemic immune-inflammation index; SIRI, Systemic inflammatory response index; PNI, prognostic nutritional index; APACHE II, Acute Physiology and Chronic Health Evaluation II; SOFA score, sequential organ failure assessment score; WBC, white blood cell; MCV, mean corpuscular volume; MPV, mean platelet volume; AST, aspartate aminotransferase; ALT, alanine aminotransferase; LDH, lactate dehydrogenase; LDL-C, low molecular weight lipoprotein cholesterol; HDL-C, high molecular weight lipoprotein cholesterol; CRP, C-reactive protein; PRC, procalcitonin; TSH, thyroid stimulating hormone; INR, international normalized ratio; LOS, length of stay; ICU, intensive care unit. Rho coefficients are presented in the table. Significant rho coefficients are highlighted in bold; *denotes P<0.05, and **indicates P<0.01.
